# Lingual Ultrastructural and Histochemical Study in the Patagonian Mara (Rodentia: Caviidae, *Dolichotis patagonum*) in Relation to Other Hystricomorphs

**DOI:** 10.3390/ani13243889

**Published:** 2023-12-18

**Authors:** Petr Čížek, Karolina Goździewska-Harłajczuk, Pavla Hamouzová, Joanna Klećkowska-Nawrot, Pavel Kvapil

**Affiliations:** 1 Department of Anatomy, Histology and Embryology, Faculty of Veterinary Medicine, University of Veterinary Sciences Brno, 612 42 Brno, Czech Republic; cizekp@vfu.cz; 2 Department of Biostructure and Animal Physiology, Faculty of Veterinary Medicine, Wrocław University of Life Sciences, 51-631 Wrocław, Poland; joanna.kleckowska-nawrot@upwr.edu.pl; 3 Department of Physiology, Faculty of Veterinary Medicine, University of Veterinary Sciences Brno, 612 42 Brno, Czech Republic; hamouzovap@vfu.cz; 4 Ljubljana Zoo, 1000 Ljubljana, Slovenia; pavel.kvapil@gmail.com

**Keywords:** herbivorous diet, histochemistry, hystricomorph, lingual papillae, lingual prominence, salivary glands

## Abstract

**Simple Summary:**

The aim of this study was the analysis of the lingual surface and lingual glands of the Patagonian mara’s tongue. The tongues were collected from three *Dolichotis patagonum* from Ljubljana zoo. Light microscopy (H&E, Masson–Goldner, and Azan trichrome staining methods) and ultramicroscopy were used for the description of the research material features. The analyses of the lingual gland secretions were performed with PAS, AB pH 2.5, AB pH 1.0, PAS/AB pH2.5, and HDI staining methods. Some typical signs of adaptation to herbivorous diet (e.g., a well-developed lingual prominence) were found. The type of the secretion of the lingual glands with other features of the lingual surface confirm the adaptation of the Patagonian mara to grass-feeding.

**Abstract:**

The study describes the ultrastructure of the tongue in the Patagonian mara (*Dolichotis patagonum*) using light and scanning electron microscopy. Histochemical methods were used for evaluation of lingual salivary glands. The tongue is divided into a small and rounded apex, a narrow corpus, and a significantly wider radix, with a well-developed lingual prominence. The tip of the apex is free of papillae. The caudal part of the apex and the corpus are covered by filiform papillae. Round fungiform papillae are scattered among them. Papillae on the narrow stalk are conical. The radix contains caudally bent papillae forming wide flat prominences, slender, hook shaped filiform papillae, foliate papillae, and two oval vallate papillae. Taste buds were found on the lateral sides of the foliate and vallate papillae. Purely serous salivary glands are beneath the vallate and foliate papillae. Serous acini and mucous tubules are in the lingual radix. The Patagonian mara is the only hystricomorph rodent with described hyaline cartilage strengthening the lingual radix. Some typical signs of adaptation to herbivorous diet were found. The structure of the tongue is adapted to grass-feeding, as grasses form the main component of their diet.

## 1. Introduction

The Patagonian mara (*Dolichotis patagonum*) is a rodent, a member of the family Caviidae and suborder Hystricomorpha. Hystricomorph rodents are a monophyletic group and share a comparable biogeographic history with New World monkeys (Platyrrhini). It has been suggested that both groups dispersed to South America in a single colonization event from Africa [1,2]. *Dolichotis patagonum* is endemic to Argentina across the Monte and Patagonian drylands, and lives in flat, open areas with heterogeneous vegetation structure [3]. This species is considered Near Threatened according to the IUCN Red List [4]. Maras are herbivores, feeding mainly on grasses (predominantly *Stipa* spp., *Poa* spp., *Setaria* spp.) and shrubs (*Prosopis* spp., *Chuquiraga* spp.) [5,6]. All hystricomorph rodents are strict herbivores, feeding mainly on leaves. A detailed description of their eating habits is given in Table 1.

Previous scanning electron microscopy studies of the ultrastructure of the tongue in Hystricomorpha have described mechanical filiform and conical papillae, and gustatory fungiform, vallate, and foliate papillae in most species [7,8,9,10,11,12], including the Patagonian mara [13]. Foliate and fungiform papillae lack in the degu, *Octodon degus* [14]. The localization of taste buds was thoroughly described in the tongue surface in some Hystricomorpha [7,12,15]. A description of connective tissue cores (CTC) was performed in some hystricomorphic species [7,8,9,11,12], as well as a detailed description of lingual salivary glands [7,8]. However, to our knowledge, histochemical analysis of the lingual salivary glands in Hystricomorpha has not yet been performed. Therefore, this information is still lacking.

The aim of this study is to describe the lingual ultrastructure in Patagonian mara in relation to its herbivorous feeding habits and to compare the results with other hystricomorphic rodents. Since scanning electron microscopy of the dorsal lingual surface has already been performed (see [13]), this study is also focused on the histochemical analysis.

**Table 1:** Diet in selected hystricomorph rodents.

Species	Familia	Feeding Characteristics
Patagonian mara (*Dolichotis patagonum*)	Caviidae	Herbivores feeding mainly on grasses and shrubs [5,6]
guinea pig (*Cavia porcellus*)		Strict herbivores feeding on many kinds of vegetation [16,17]. In captivity, they feed on grass, hay, pellets and vegetables [16].
capybara (*Hydrochoerus hydrochaeris*)		The diet consists mainly of grasses, also aquatic plants, grains, melons, and squashes [17].
rock cavy (*Kerodon rupestris*)		Herbivores feeding on leaves, shoots, branches, fruits, tree barks, roots, and tubers of the vegetation [18].
degu (*Octodon degus*)	Octodontidae	Foliovores, granivores and lignivores. They feed on leaves, bark, stems and seeds of shrubs and forbs. They prefer young leaves and avoid woodier shrubs [19].
porcupine (*Hystrix cristata*)	Hystricidae	Feeding on bark, roots, tubers, rhizomes, bulbs, fallen fruits, and cultivated crops [17].
agouti (*Dasyprocta aguti*)	Dasyproctidae	Herbivores feeding on fruit pulp, seeds [7] and vegetables [17].
nutria (*Myocastor coypus*)	Myocastoridae	The diet is strictly vegetarian [17], includes monocots associated with water (40–60%), terrestrial monocots (30–35%), and dicots (0–15%) [20].
chinchilla (*Chinchilla lanigera*)	Chinchillidae	Herbivores feeding on leaves (dried rather than fresh), roots, fruit, berries, bark, alfalfa, grasses, shrubs, and cacti. The diet is naturally high in fibre coming from bark, woody stems, and bromeliads [16].

## 2. Materials and Methods

All Patagonian maras whose tongues were used in this study were kept in the zoological collection of Ljubljana Zoo (Slovenia) and died naturally or were euthanized at the veterinary ambulance of Ljubljana Zoo due to serious injuries. The cause of euthanasia did not affect the oral cavity. No experimental procedure was performed on the animals; only the material from naturally died or euthanized animals was collected. The tongues of three adult (one male and two females) maras were used in this study. All samples were fixed in 10% neutral buffered formalin.

The samples for light microscopy were processed according to routinely used laboratory methods (dehydration in graded ethanol series, immersion in xylene, infiltration with hot paraffin, and embedding in paraffin blocks) [21]. The slides were stained with haematoxylin and eosin (H&E), Masson–Goldner trichrome (MG trichrome), and Azan trichrome methods for histological analysis. Other sections were stained using Alcian blue pH 1.0 (AB pH 1.0), Alcian blue pH 2.5 (AB pH 2.5), Hale’s dialysed iron (colloidal iron; HDI), periodic acid Schiff (PAS), and PAS/AB pH 2.5 [22,23] for histochemical analysis. The stained samples were analysed using the Zeiss Axio Scope A1 light microscope (Carl Zeiss, Jena, Germany) based on the Spicer and Henson (1967) methodology [24].

The samples for scanning electron microscopy were dehydrated in a graded alcohol series (30%, 50%, 70%, 80%, 90%, 96%, and 100%, 3 × 10 min for each concentration), transferred to absolute acetone, dried at the critical point (Bal-tec CPD 030 Critical Point Dryer, Bal-Tec, Reading, UK), coated with gold (Balzers SCD 040 by current 30 mA for 4 min), and finally examined and photographed under a Tescan VEGA TS 5136 XM scanning electron microscope (TESCAN, Kohoutovice, Czech Republic) in a high vacuum and accelerated voltage 20 kV by using an SE detector (TESCAN, Kohoutovice, Czech Republic).

All anatomical and histological terms were based on the terminology of *Nomina Anatomica Veterinaria* (2017) [25] and *Nomina Histologica Veterinaria* (2017) [26]. As these two sources include terminology related exclusively to domestic animals, the terms in general use, which are firmly used for some wild animals, were also given. In these cases, both terms were given and the appropriateness of using the term is discussed in the discussion.

## 3. Results

### 3.1. General Morphology

**Figure 1:**
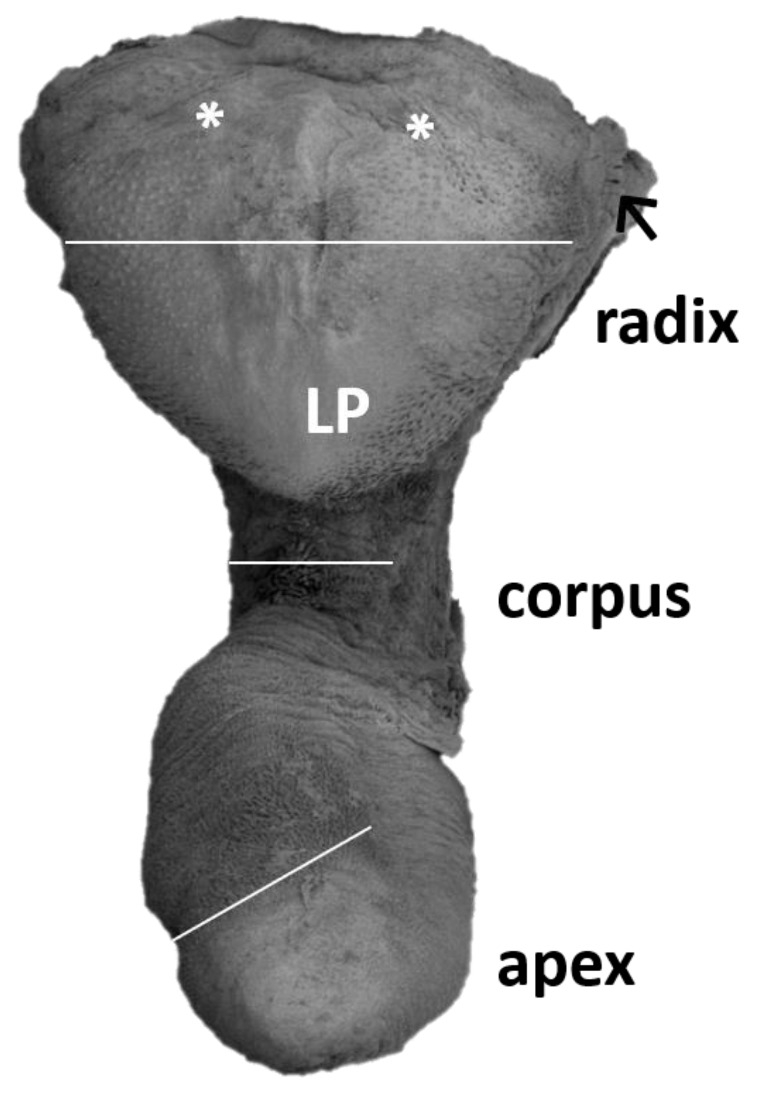
General overview of the tongue of the Patagonian mara. Foliate papillae (arrow), vallate papilla (*), LP—lingual prominence. The corpus is narrowed. The radix rises to a high lingual prominence. White lines indicate areas of the size measurement.

The tongue of the Patagonian mara is divided into three parts: a small and rounded apex, a narrow corpus, and a significantly wider radix, with a well-developed lingual prominence. A median groove (*sulcus medianus linguae*) is not formed (Figure 1). The average size was 6.7 cm (length), 0.4 cm (apex width), 1.4 cm (corpus width), 2.6 cm (radix width), 0.9 cm (height), and 2.0 cm (lingual prominence height). The shape of the tongue does not differ between males and females.

### 3.2. Lingual Apex

**Figure 2:**
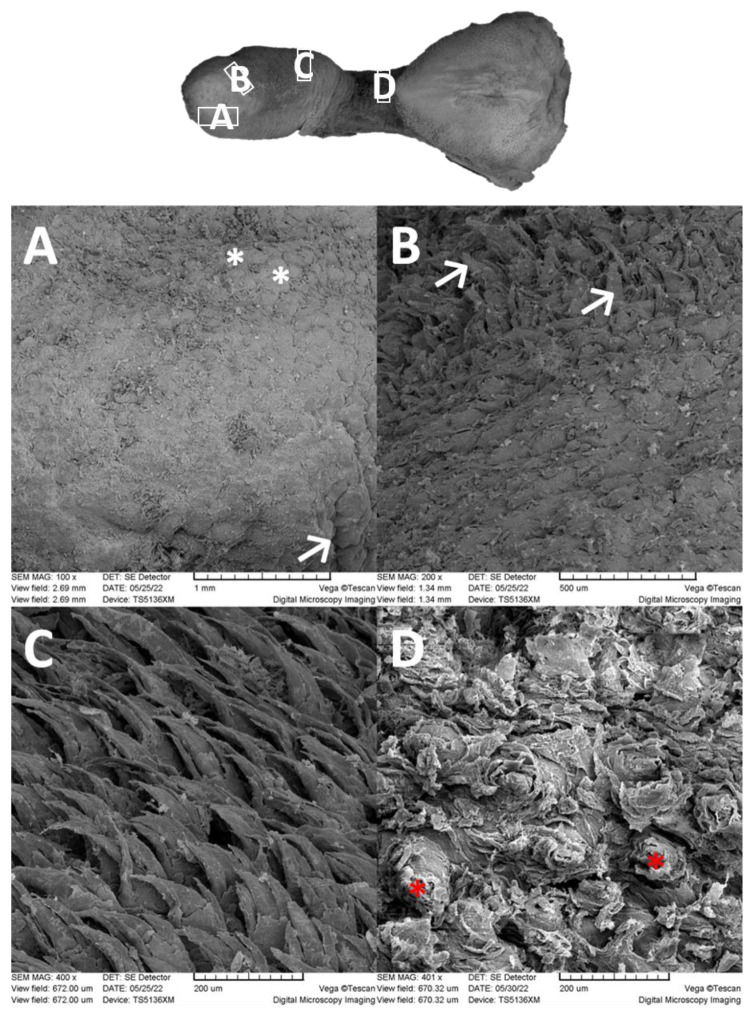
Scanning electron microscopic views of the papillae in the apex and corpus. (**A**): Flat prominences (*) covering the tip of the apex. Thick ridges are on the lateral surface (arrow). (**B**): Filiform papillae on the dorsal caudal part of the apex (arrows). (**C**): Filiform papillae in the corpus are bent caudally and consist of primary papilla and one or more secondary papillae. (**D**): Conical papillae with a wider base (*) on the narrow stork of the corpus. The desquamation is obvious.

**Figure 3:**
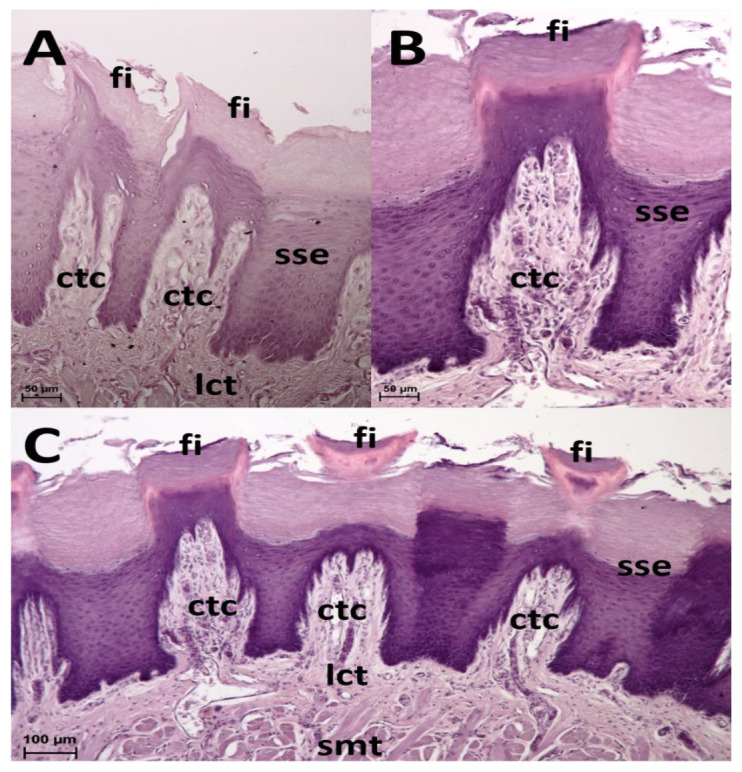
Mechanical papillae. (**A**): Filiform papillae (fi) in the lingual apex are covered by stratified squamous keratinised epithelium (sse) covering the connective tissue cores (ctc) extending from the loose collagenous connective tissue layer (lct). HE staining. (**B**): Filiform papillae (fi) in the lingual corpus are covered by stratified squamous keratinized epithelium (sse) covering the connective tissue cores (ctc). HE staining. (**C**): Filiform papillae (fi) in the lingual corpus are covered by stratified squamous keratinized epithelium (sse) covering the connective tissue cores (ctc) extending from the loose collagenous connective tissue layer (lct), under which the skeletal muscle tissue (smt) is arranged in various directions. HE staining.

The small lingual apex is generally dome-shaped. Its papillae-free tip is covered by flat prominences that dorsally and caudally slightly increase in height to form rather rounded filiform papillae. These papillae tend to extend in several short projections. On the lateral surface of the lingual apex, irregularly outlined thick ridges are formed (Figure 2A,B). The epithelium of the lingual apex is stratified squamous and highly keratinized (Figure 3A).

### 3.3. Lingual Corpus

The lingual corpus is a gradual continuation of the lingual apex. However, before it reaches the lingual radix, it narrows significantly to form a stalk-like connection (Figure 1). The rostral wider part represents approximately two thirds of the length of the corpus, whereas the caudal narrow stalk represents the remaining one third. The dorsal lingual surface is covered by a uniform population of filiform papillae. They appear as caudally bent slender processes that extend in one major projection (primary papilla) and often also in the form of one or more smaller secondary papillae (Figure 2C). Round fungiform papillae are scattered among filiform papillae on the dorsolateral surface. Papillae on the narrow stalk are different. Because of their wider base they are classified as conical papillae (Figure 2D). The stratified squamous epithelium is highly keratinized. The desquamation is more evident caudally in the area of the stalk (Figure 3B,C).

### 3.4. Lingual Radix

**Figure 4:**
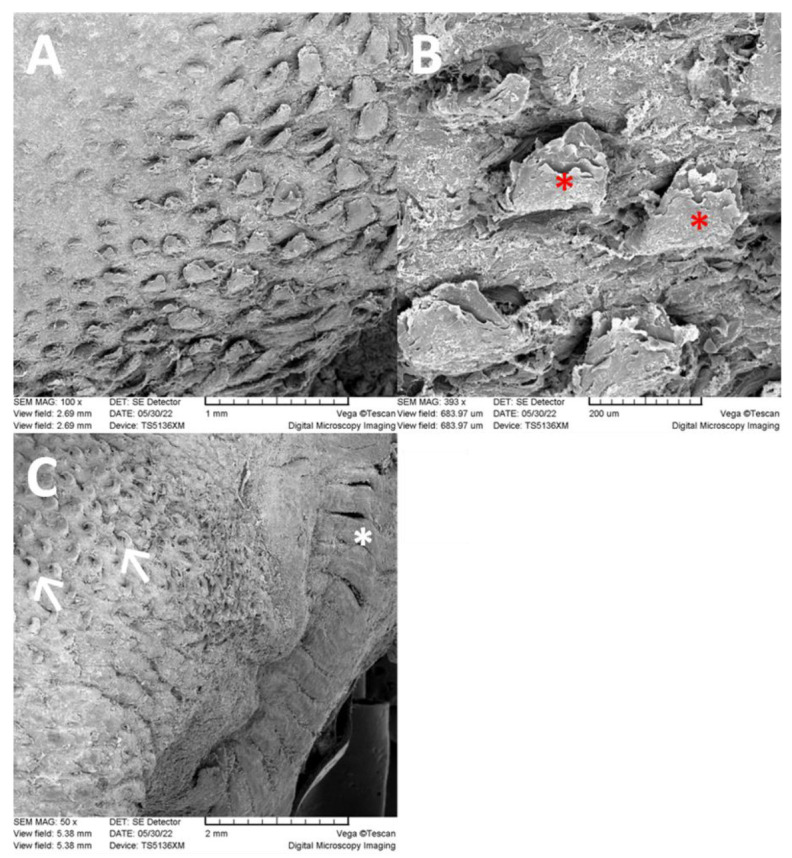
SEM images of the papillae in the radix. (**A**): Layout of filiform papillae on the dorsolateral surface of the radix. (**B**): Filiform papillae of the radix appear as caudally bent wide flat prominences (*). Desquamation is evident around the papillae. (**C**): Slender, hook-shaped papillae (arrows) and foliate papillae (*) on the lateral surface.

**Figure 5:**
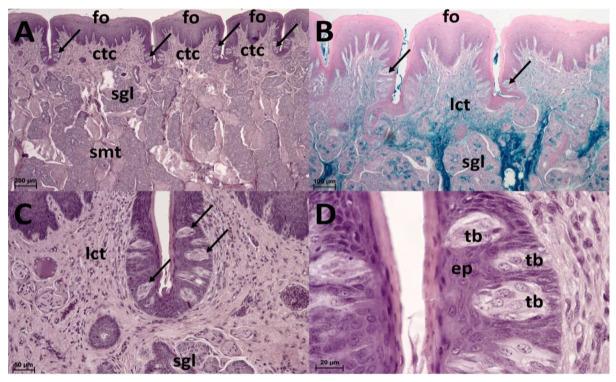
Foliate papillae. (**A**): Foliate papillae (fo) are covered by stratified squamous epithelium (sse) covering the connective tissue cores (ctc). Serous glands (sgl) are beneath the papillae. Skeletal muscle tissue (smt) extends among them. Taste buds (arrows) are at the bases of the foliate papillae. HE staining. (**B**): Foliate papillae (fo), loose connective tissue (lct, green colour), serous glands (sgl), taste buds (arrows). HDI staining. (**C**): Taste buds (arrows) in the stratified squamous epithelium at the bases of foliate papillae. HE staining. (**D**): Detail of the epithelium (ep) with taste buds (tb). HE staining.

**Figure 6:**
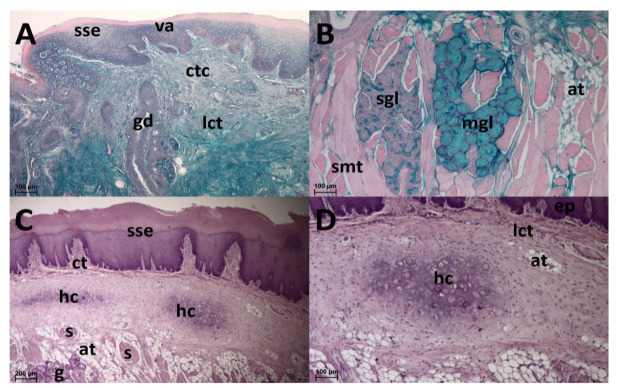
Lingual radix. (**A**): Vallate papilla (va) consists of the connective tissue core (ctc) covered by the layer of stratified squamous epithelium (sse). Glandular ducts (gd) are found in the loose collagenous connective tissue (lct) in its proximity. AB pH 2.5 staining. (**B**): Salivary glands. Serous acini (sgl) and mucous tubules (mgl) among the fascicles of skeletal muscle tissue (smt). Among them, white adipose tissue (at) occurs. HDI staining. (**C**): Areas of hyaline cartilage (hc) are found in the lingual radix. Stratified squamous epithelium (sse), loose connective tissue (ct), skeletal muscle (s), white adipose tissue (at), salivary glands (g). HE staining. (**D**): Detail of the hyaline cartilage (hc). Surface epithelium (ep), loose collagenous connective tissue (lct), white adipose tissue (at). HE staining.

The lingual radix contains a large heart-shaped lingual prominence (Figure 1). It is papillae free medially in the area of its tip. However, on the lateral surface of the tip there are papillae found. They appear as wide flat prominences that are bent caudally. In the dorsal and caudal direction, the number of these papillae decreases so they are only sparsely distributed. The lingual surface among these papillae is smooth (Figure 4A,B). Caudally, the lateral surfaces of the lingual prominence bear filiform papillae. These papillae are slender, hook shaped, and more numerous than the papillae located rostrally on the tip of the lingual prominence. At the caudal end of the lingual radix, the filiform papillae cease to appear and instead, irregularly shaped ridges cover the dorsal lingual surface. These ridges contain copious openings of the glandular ducts. Epithelium of the lingual radix is again stratified squamous. However, the desquamation is least evident. On the lateral surface of the lingual radix, the foliate papillae extend throughout its whole length. They appear as long vertically oriented strips. Taste buds are in the stratified squamous epithelium at the bases of foliate papillae (Figure 5). Two oval vallate papillae are incompletely surrounded by the circumpapillary sulcus (*sulcus papillae*) in the form of lateral surfaces of the papillae. Taste buds are in the stratified squamous epithelium of the lateral sides of the vallate papillae (Figure 6A). The lingual radix is strengthened with hyaline cartilage (Figure 6C,D).

Purely serous salivary glands (*glandulae linguales gustatoriae*) are beneath the vallate and foliate papillae. Serous acini and mucous tubules are among the fascicles of skeletal muscle tissue in the lingual radix; serous glands are more superficial than the mucous glands. Serous and mucous glands are separated by fascicles of skeletal muscle tissue. No mixed glands are at their interface, and the transition of individual types is sharp (Figure 6B).

**Figure 7:**
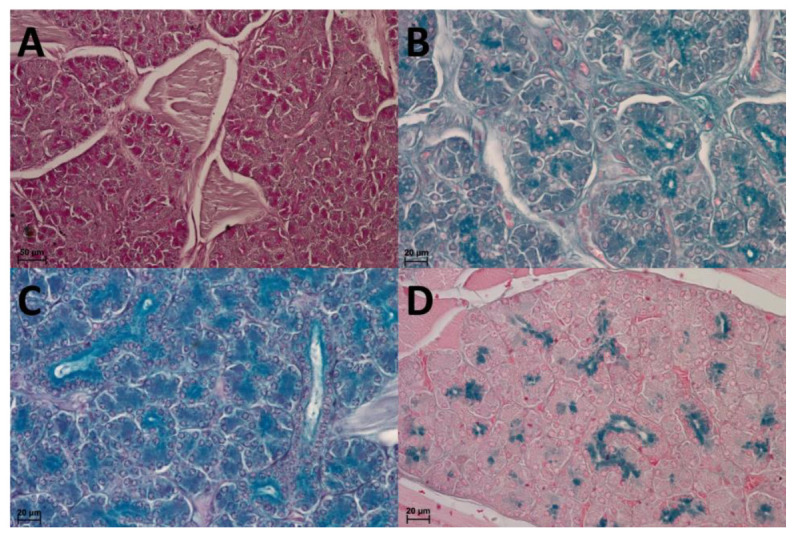
Serous salivary glands in the lingual radix. (**A**): Serous acini with weakly positive reaction (+)—mild magenta colour. PAS staining. (**B**): Weakly positive reaction (+) in some serous acini (light blue). AB pH 2.5 staining. (**C**): Positive reaction (++)—blue colour in some of acini cells. AB pH 2.5 PAS staining. (**D**): Weakly positive reaction (+)—light blue colour in some serous acini; while domination of negative reaction (−) within most of the serous acini. HDI staining. PAS staining.

**Figure 8:**
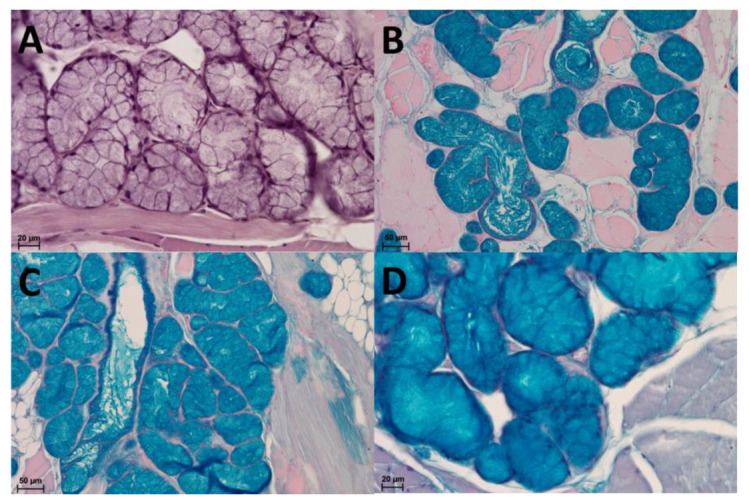
(**A**): Mucous acini in HE staining. (**B**): Mucous acini with strong positive reaction (+++)—dark blue colour. AB pH 1.0 staining. (**C**): Mucous acini with strong positive reaction (+++)—dark blue colour. AB pH 2.5 staining. (**D**): Positive reaction (++)—blue colour in mucous acini. PAS-AB pH 2.5 staining.

The AB pH 2.5 staining confirmed a strong positive reaction in mucous acini and a weakly positive reaction in serous acini. The PAS-AB pH 2.5 staining showed positive reaction (blue) in mucous acini, while weakly positive reaction in serous acini (dark blue) in some cells, which confirms the presence of secretion containing combinations of both acidic and neutral glycoconjugates. The AB pH 1.0 staining positive reaction confirms the presence of sulphated glycoconjugates. A weakly positive reaction (+) was also in PAS staining of mucous acini. The weakly positive PAS reaction confirms the sparse presence of neutral glycoconjugates in mucous acini or serous acini (Figure 7 and Figure 8).

## 4. Discussion

The tongue has a similar shape (a narrow corpus and a wide radix) in various hystricomorph rodents [7,8,18]. The presence of a lingual prominence was described in *Dolichotis patagonum*, *Cavia porcellus* [8], *Kerodon rupestris* [18], and *Dasyprocta aguti* [7]. However, the lingual prominence is not a typical structure of hystricomorph rodents, as it was also described in some myomorph rodents [27,28,29] or castorimorph rodents [30]. In sciuromorph rodents, it was not developed [31] or was inconspicuous [32]. Many other graminivores or folivores belonging to the order Artiodactyla or Perissodactyla have a lingual prominence. Lingual prominence has been identified in two of three families of perissodactyls, Rhinocerotidae [33] and Tapiridae [34], as well as in many ruminant or tylopod artiodactyls regardless of whether they were browsers or grazers [35,36,37,38,39,40,41,42,43,44,45,46,47,48,49,50,51,52,53,54,55,56,57,58,59,60,61,62,63,64,65,66,67,68,69,70,71,72,73,74,75,76,77,78,79,80], and in *Hippopotamus amphibius*, a non-ruminant herbivorous artiodactyl [81]. To our knowledge, an absence of the lingual prominence was not described in any bovid, cervid, or giraffid ruminant. However, the lingual prominence was not developed in *Tragulus javanicus*, Tragulidae [82]. The lingual prominence is not developed in equids [25,83,84] and in some folivores belonging to Marsupialia or Pilosa [85,86]. Thus, the lingual prominence occurs in various herbivores across many orders, but it is not developed in some species with herbivorous diet. Carnivorous and omnivorous monkeys do not have a lingual prominence. Pigs are omnivorous artiodactyls and they do not have a lingual prominence [80]. The lingual prominence appears to be a characteristic structure that has developed primarily in grass-eating animals [80]. It is considered a typical sign of adaptation to a herbivorous diet [30].

The lingual prominence is very similar to the *torus linguae*, a structure typical of ruminants. *Torus linguae* is the correct term in domestic animals [25]. However, by searching previous studies, we found that the term lingual prominence is greatly preferred in non-ruminants. Perhaps this is because *Nomina Anatomica Veterinaria* specifically states that the term refers to ruminants, so the authors were reluctant to use it for other animal species. Due to the enormous number of studies in which the term lingual prominence is used in non-ruminants, we do not dare to replace it with the term *torus linguae*, even if the differences are minimal.

Tip of the lingual prominence rising above the body of the tongue is a place of the side-to-side separation of collected food in the direction of the teeth and on the surface of the lingual prominence [62]. In ruminant grazers, the lingual prominence is typically covered with conical papillae [38,62,80]. In *Dolichotis patagonum*, conical papillae are on the corpus, but are replaced by caudally bent wide flat filiform papillae on the radix. The large conical papillae located on the lingual prominence are mechanically effective for repeated mastication of grasses in the oral cavity of ruminants [80], but they are not found on the radix of non-ruminant herbivores [38]. Thus, the absence of conical papillae in this area of the Patagonian mara tongue is not surprising. The lateral surface of the tip of the lingual prominence contains wide flat prominences that are bent caudally. Caudally, the lateral surfaces of the lingual prominence bear numerous slender, hook shaped filiform papillae. The caudally directed filiform papillae participate in the transport and swallowing of food [38]. In *Dolichotis patagonum*, the filiform papillae are located on the lateral areas of the lingual prominence, while the medial surface is smooth. This was not described in other hystricomorphs [7,8,10,12,14]. The reason for this difference is not clear, perhaps a description of the hard palate (*palatum durum*) would help to answer this question. However, such description is not available either for the examined species or for other hystricomorphs. The food being processed is clamped between the palate and the dorsal surface of the tongue. Thus, a wrinkling of the palate mucosa could explain this finding. Further study is required for this.

All standard types of papillae were distinguished in all Hystricomorpha [7,8,9,10,11,12,18], except *Octodon degus*, which lacked fungiform and foliate papillae [14]. This species does not differ from other hystricomorphs in its feeding habits. Filiform papillae with various numbers of caudally bent projections were distributed over the whole dorsal surface [7,8,9,10,11,12,14,18]. Conical papillae were not mentioned in *Chinchilla lanigera* [11] and *Hydrochaeris hydrochaeris* [12], but were described in all other hystricomorphs [7,9,10,14,18]. Mechanical papillae ranged between conical and branched in *Cavia porcellus* [8]. Fungiform papillae of hystricomorphs were dome-shaped [7,8] or round [11,13]. However, their distribution varied. They were scattered among filiform papillae in *Dolichotis patagonum*, *Chinchilla lanigera* [11], and *Myocastor coypus* [9]. In addition to this distribution, rows of fungiform papillae were on the lateral margins in *Cavia porcellus* [8] and *Hydrochaeris hydrochaeris* [12]. No fungiform papillae were in the apex in *Dolichotis patagonum*, while they were abundant in the tip of the tongue in *Chinchilla lanigera* [11]. Unlike in most hystricomorphs, the gustatory papillae were found only in the corpus and radix in *Dolichotis patagonum*. Thus, their sensory abilities at the tip of the tongue are very limited, similarly as in *Octodon degus* [14]. Two vallate papillae were found in all hystricomorph rodents [8,9,10,11,12,13,14,18] except *Dasyprocta agouti*, which had four of them [7]. The authors [7] did not state possible rationales, since the morphological studies of the lingual papillae of rodents, suborder: Hystricomorpha, were limited. In all hystricomorphs except *Octodon degus*, foliate papillae were on the edges of the radix and differ in the number of ridges from 5 in *Cavia porcellus* [8] to 20 in *Hystrix cristata* [10].

Not all studies included a description of lingual glands. Moreover, the description of the glands was incomplete in some species. Serous glands located near vallate papillae were described in all hystricomorph rodents in which the light microscopy structure of the tongue was analysed [7,8,10,12,14,18]. Mucous glands were distinguished only in the proximity of the foliate papillae in *Cavia porcellus* [8] and deeper in the radix in *Hystrix cristata* [10] and *Octodon degus* [14]. In Patagonian maras, purely serous glands were found under the lingual surface of the radix, whereas purely mucous glands were deeper, similarly as was described in *Hystrix cristata* [10] and *Octodon degus* [14]. The mucous secretion aids in swallowing dry food and facilitates tongue movement, the serous secretions in the tongue are suggested to be involved in taste perception by washing out the food substances from the taste pores of gustatory papillae and dissolving food elements and distributing them to taste buds [31].

The histochemical profile of the lingual salivary glands may be related to the diet [87]. Histochemical analysis of the lingual salivary glands has not yet been performed in other hystricomorph rodents. Therefore, the only description is based on our results. This study revealed the presence of secretion containing combinations of both acidic and neutral glycoconjugates, the presence of sulphated glycoconjugates and the sparse presence of neutral glycoconjugates in mucous or serous acini. Acidic carbohydrates with sulphated acid and a few neutral mucins were reported in *Sciurus anomalus* [31] and *Jaculus jaculus* [88], acidic as well as neutral mucopolysaccharides in rat, mouse and hamster [89]. The secretion of von Ebner’s glands in the Wistar rat did not contain neutral mucins, but other types of PAS-positive substances, whereas Weber’s gland synthesized both PAS-positive neutral mucins, and Alcian blue-positive acidic mucins [90]. In *Spalax leucodon*, the mucous cells were rich in Alcian blue positive mucosubstances, but the PAS mucosubstances showed very weak reaction, and this mucosubstances were present at a very less amount in serous cells. In the PAS/AB staining, the serous and mucous cells showed only Alcian blue or only PAS reaction [91]. In the sheep (*Ovies aries*), the serous glands showed moderate intensity of PAS reaction and strong intensity of Alcian blue. It had intense and weak reaction for sulphated and acid mucopolysaccharides respectively. In the goat (*Capra hircus*), the mucous glands had strong PAS reaction and were Alcian blue-negative. Sulphated acid mucopolysaccharides were negative. More sulphated acid mucopolysaccharides was present in sheep than in goat [92].

Hyaline cartilage strengthening the lingual radix was described only in the Patagonian mara, not in any other hystricomorph rodent. We cannot suggest the reason for its development in mara, as the feeding habits and tongue use are not different from its relatives. The cartilage probably serves to strengthen the tongue. Compared to leaves, grasses are more resistant to chewing [93]. This may be related to the strengthening of the root of the tongue, because maras feed mainly on grasses. The horse, a species in which the lingual radix is known to be strengthened by the cartilage [25], also belongs to grazers [94]. Although *cartilago dorsi linguae* is generally developed in *Equus caballus* [25], its absence was described in the tongue of Caspian miniature horse [95]. In *Equus asinus*, *cartilago dorsi linguae* was observed in 10% individuals only [96]. As differences in the presence of the lingual cartilage occur even within one genus (*Equus*), and there are also individual differences [96], it is not surprising that these differences emerged within the suborder. *Dolichotis patagonum* is the only known member of this subspecies in which the lingual cartilage occurs, but it cannot be ruled out that cartilage will also be found in other hystricomorphs whose tongues have not yet been described.

## 5. Conclusions

Hystricomorph rodents are a monophyletic group [1]. Moreover, all the members of this suborder have similar feeding habits (as was described in Table 1). Therefore, the morphology of the tongue does not differ much within hystricomorph rodents. Some typical signs of adaptation to herbivorous diet (e.g., a well-developed lingual prominence [30]) were found. The structure of the tongue of Patagonian mara is adapted to grass-feeding, as grasses form the main component of their diet.

The obtained results will contribute to the knowledge of the microscopic structure of the tongue in strictly herbivorous rodents and will be helpful in future comparative studies and research on the species adaptation to the type of diet.

## Data Availability

The data presented in this study are available on request from the corresponding author.
